# Medical Opinion and Movement

**Published:** 1909-04-10

**Authors:** 


### Medical Opinion and Movement.

THE Hunterian lecture on X-ray Carcinoma before the Royal College of Surgeons by Mr. C. W. Rowntree sums up our present knowledge of its incidence and the results, so far, of experimental inquiry. He emphasises the low degree of malignancy of these forms of cancer, and the comparative youth of most of the sufferers. As bearing on the question whether the effect is merely that of long continued irritation, strictly comparable with the causes of epithelioma in sweep's warts, paraffin eczema, Paget's eczema, and so on, or whether there is some specific action of the rays, attention is called to the multiple nature of the growths in five out of the eleven cases of *x*-ray cancer now reported in this country, whereas in the other precancerous conditions enumerated any resulting malignant growth is practically always single. By experiments on animals it seems to be established that the first effect of the rays in doses insufficient to cause dermatitis and burns is a stimulant one: and that when long continued this is exchanged for a destructive action, which is, however, to a certain extent selective; that is, it affects certain tissues more than others. The action of the rays upon the hairy scalp and upon the secreting cells of the testis is itself evidence of such selection. The importance of a clear perception of the cell-activating properties of small doses is made plain by the lecturer's suggestion that possibly the disappointing results in many cases of epithelioma treated by *x*-rays may be due to stimulation of the deeper parts of the growth coincident with destruction of the more superficial parts.

IN the Long Fox memorial lecture delivered at Bristol, Mr. J. H. Parsons chose as his theme the Metastatic Inflammations of the Eye. After discussing the ophthalmic manifestations of general blood infections, as by streptococci, gonococci, and so on, he dealt with the pathology of two of the most obscure diseases of the eye, iridocyclitis and sympathetic ophthalmia. Mr. Parsons holds that iridocyclitis, even when apparently idiopathic, is really a result of metastatic inflammation. Pyorrhœa alveolaris is the commonest primary focus; failing that he searches for suppuration in the nasal sinuses, the genital organs, or, failing these, the alimentary canal. In support of the last possibility he finds that many cases do well on calomel and other intestinal antiseptics, and suggests the lactic acid bacillary treatment. As for sympathetic ophthalmia, he remarks that it has all the characters of a disease due to bacteria, and that the failure hitherto to discover the specific organism is no proof of the contrary. The migratory theory of transmission by way of the optic nerves and chiasma is now abandoned, and the lecturer prefers the metastatic theory of infection by the blood. He explains the selection of the opposite eye for the secondary outbreak by supposing the organism to be only pathogenic to the eye, because there alone does it find a suitable nidus, and only then if the conditions of tissue resistance are favourable. Prolonged latent period is accounted for by encapsulation in the exciting eye or, in rare cases, in other organs of the body. He considers that the organism undergoes development in the exciting eye, thus accounting for the usual latent period. The conception is an interesting one.

THE Cause of Fatigue was recently discussed by Sir Lauder Brunton during a debate at the Medical Society reported in the *Clinical Journal*. The quickened pulse and respiration were until lately regarded as chiefly of mechanical origin, but Sir Lauder now adopts the views of Mosso as to the production of definite toxins by the muscles during exertion. Thus he has found that if a narcotised dog be transfused with blood from a dog which has been at rest, no effect is produced upon the pulse and respiration; when, however, that of an animal which has been tetanised is employed, acceleration of both rates is produced just as if the narcotised dog had itself been tetanised. From this conception he passes on to that of fatigue antitoxins, which are, he believes, produced in the system in response to the fatigue toxins, just as antivenims in response to small doses of snake venom, and diphtheria antitoxin when diphtheria bacilli are used. The principles so well known of the immunising effect, owing to the formation of these antibodies, of small commencing doses, gradually increased, are utilised to explain the phenomena of the familiar process of "training." The athlete begins his course of training, if he is wise, with quite gentle exercise, thus auto-inoculating himself with fatigue toxins and causing an output of corresponding antitoxins. Gradually he increases his dose of fatigue, at the same time multiplying enormously his capacity for making fatigue antibodies; and thus he is able after a few weeks' training to endure exertion, that is to say, to neutralise toxins, by which he would when untrained be completely prostrated.

BONI contributes to a recent number of the *L'Ospedale Maggiore* a *résumé* of cases of Early Typhoid Fever with a view to throwing light on the diagnosis of this condition. Taking a very large series of cases, he found that the most positive evidence is furnished by blood examinations, regularly and systematically undertaken. This was positive in no less than 92 per cent. of cases. The other signs which are of great value in furnishing diagnostic data are the typhoid tongue, on which he lays stress, as it was shown in 90 per cent. of cases, enlargement of the spleen (85 per cent.), the diazo-reaction (83 per cent.), and headache (80 per cent.). Lower down in value, and in order of their relative importance, he places albuminuria, diarrhœa, ileocæcal gurgling, serum diagnosis, typhoid spots, a dicrotic pulse, and a slow pulse rate in relation to the temperature. None of these, however, can outweigh the value of a regular blood examination, to which he attaches the greatest importance.

THESE findings are of interest in view of the fact that in private practice the early diagnosis of typhoid fever is of the greatest importance, and it is not always easy for the practitioner to avail himself of the services of an expert bacteriologist. Under these conditions he has to depend largely on an examination of the patient, and to base his diagnosis on such points as he can make out from that examination. The diazo-reaction, which has of late years been almost disregarded as a diagnostic factor in this disease, is well worth further study, and to the practitioner at least it should prove a valuable test when taken in conjunction with other signs. Alone its value is, of course, exceedingly doubtful, since it is now well recognised that it is given in many other conditions, and is, in fact, present whenever there is any bacterial infection of the blood. It is, however, a striking testimony to the value of this reaction that the author should place it so high in his list of diagnostic signs—much higher, in fact, than he places serum diagnosis. Boni disregards the other early signs on which some authorities have recently laid stress, such as the yellow pigmentation on the palms of the hands. According to him they are too inconstant and too vague to be of great diagnostic value, and when they are present one or more of the other signs are usually to be found present as well on close examination.

ATTENTION has been already drawn in these columns to the method adopted by certain surgeons on the Continent of allowing patients to get up within three or four days after abdominal operation. Dr. Schücking, writing in the *Zentral-Blatt für Gynäkologie*, advocates in preference gymnastic exercises in bed. They should be carried out at first for half an hour twice a day and should be specially directed to the use of the muscles of the thorax and abdomen. The pulse and blood-pressure should be watched, and the exercises regulated accordingly. The author has used the method especially for patients after labour, and after gynæcological operations, but he has also found these exercises in bed very effective in cases of anæmia with feeble heart action and in cases of dilated heart. They improve the tone of the general system as well as strengthening the muscles, and by activating the circulation they tend to prevent the occurrence of thrombosis and embolism. It certainly seems more rational to innervate the general muscular system by such exercises if it be deemed necessary or desirable after operation, than to resort to the more heroic method of allowing the patient to walk about, and, as the author points out, all the advantages claimed for the latter method are obtained by the exercises in bed without any of the inconveniences and risks which are necessarily incurred by movements in the upright position.

DR. A. L. MENTZIKOVSKY, of St. Petersburg, who has made a special study of the Incontinence of Urine in Children, has formed the opinion that the pathology of this morbid condition consists chiefly in the degree of sensitiveness, and of vascularity of the mucous membrane of the bladder and urethral canal. He distinguishes two types. In the one the mucous membrane of the urinary passages is extremely sensitive and hyperæmic. The least touch excites acute pain with intense reaction, so that it is impossible to introduce a catheter without a general anæsthetic. The second type is, on the contrary, characterised by diminished sensibility, and the interior of the bladder and urethra can be explored without causing any reaction on the part of the child. In the first case the smallest accumulation of urine in the bladder occasions a reflex contraction of the muscles and causes a continual incontinence of urine. In the second case the sensibility of the mucous membrane of the bladder is so diminished that the reflex contraction of the sphincter vesicæ is not called forth except by special volitional control, and nocturnal incontinence results. This distinction of the two types enables the appropriate treatment to be applied. In the first type of cases the author resorts to daily local applications of cocaine solution with adrenalin, first to the urethral passage and then to the bladder itself, combined with the internal administration of bromide. In this way the sensitiveness is gradually diminished and the bladder becomes accustomed to retain the urine. For the second type of case the author advocates injections of 1 to 3 per cent. silver nitrate solution twice a week, in order to increase the vascularity and sensitiveness of the parts. He thinks that the mechanical irritation by instrumentation for the introduction of the solution contributes largely to the cure.

IN this country the treatment of Enlarged Prostate by operation almost exclusively involves suprapubic prostatectomy. The excellent results obtained by Freyer and others by this method have led to its general adoption, and the old perineal route has been to a large extent discarded. At a recent meeting of the French Academy of Medicine a discussion of the subject showed a great preponderance of opinion in support of operative treatment for this condition, and the suprapubic method also found most favour. This does not, however, appear to be the case in America. In a paper by Dr. C. Eugene Lack, of Brooklyn, on the subject the different methods of treatment are reviewed, and the author shows that the perineal method of operation as carried out by Young is able to yield, at any rate in his hands, better results than have been obtained by suprapubic prostatectomy. Thus, Freyer's mortality in his first 322 cases, with 25 deaths, was 7.8 per cent., in his last 119 cases, with 9 deaths, 7.5 per cent., showing that with improved technique he has not appreciably reduced his mortality. On the other hand, Young reports 273 cases treated by his operation, which he terms conservative perineal prostatectomy, with 8 deaths or 2.8 per cent. In his last 146 cases there was only one death, and Young explains that this might have been avoided. Young claims that these cases were wholly unselected and that every case appearing for relief was operated upon, although some were already *in extremis*. As Dr. Lack puts it, the operator with the least mortality is entitled to the centre of the stage, and he unhesitatingly gives the choice, therefore, to Young's method of operation. The advantages claimed for the operation are that perfect drainage is secured, the ejaculatory ducts, the seminal vesicles, and the prostatic urethra remain intact, and the patient can control his urine two days after operation and is out of bed in three or four days. Young is of opinion that perineal prostatectomy is not only the safest operative procedure, but also much safer than the use of the catheter.

---

In the *International Journal of Surgery* Dr. Witherspoon draws attention to what he considers an error in the method of Draining the Peritoneal Cavity for general peritonitis. This error consists in draining the peritoneal cavity by means of a drain passed down into the pelvis through an incision, say, for appendicular abscess. Such a drain, the author points out, necessarily passes down between the bowel and the lateral wall of the pelvic basin. This produces adhesions of a very dense tight character between the bowel loops and the unyielding peritoneum covering the pelvic fascia, and in turn this leads to kinking or inflammatory narrowing of the bowel at this fixed point. In support of this statement the author gives details of several cases in which drainage by this method resulted in the development of symptoms of obstruction soon after the operation. These were relieved by a second operation, at which in each instance the state of things described above was found to exist. In cases of appendicitis, therefore, necessitating drainage of the general peritoneal cavity, the author advocates the following method of procedure: the usual incision is first made and the appendix is dealt with according to the circumstances and conditions of the case, and a puncture is then made just above the bladder upon the index finger of the left hand introduced through the wound. A glass tube is introduced through the puncture and carried into the pelvis, guided by the finger. This places the drain between the loops of the intestine and the posterior wall of the bladder, and does not therefore induce adhesions between the bowel and a fixed point, such as the lateral pelvic wall. It is thoroughly efficient in draining the peritoneal cavity with the patient in a good Fowler position. A second drain placed in the wound about the head of the cæcum quickly induces adhesions which close off this portion of the bowel from the general cavity and fixes the cæcum alone to the lateral wall of the false pelvis. The author claims to have had most gratifying results by this method of drainage.

---

The Sterilising Effect of the X-Rays upon the Sexual Organs has been known for some time; but until quite recently it was uncertain whether a similar influence was exerted upon the pregnant uterus. Fraenkel, of Berlin, would now seem to have settled the question by the employment of the rays in the case of a pregnant phthisical patient suffering from intractable vomiting of pregnancy, for whom ordinary surgical interference was absolutely contra-indicated by her bad general health. Twenty-five applications of the rays for periods of from five to ten minutes each, without other interference, whether by vaginal examination or surgical procedure, resulted in spontaneous expulsion of the fœtus, accompanied by pain and rather severe bleeding, which latter, however, stopped when the uterus had expelled its contents. The author recognises the folly of trying to evolve a general rule from a single fact; but if his observations are confirmed by subsequent workers, yet another use may have to be added to the already long list of therapeutic applications of the *x*-rays. One cannot, however, avoid the conclusion that if the rays really possess this property a powerful weapon, and one which, if skilfully employed, no physical examination could possibly detect, will be placed in the hands of the criminal abortionist.

---

Le Filliatre claims to have demonstrated the superiority of Intraspinal Injections of Cocaine over similar injections of stovaine, and in addition proposes to substitute such injections for general anæsthesia by chloroform, etc., in most operations, including those on the head and neck. We have already devoted much space to this and kindred topics; but since the principles and practice of spinal analgesia are still very much *sub judice*, it is important to record the views and results of all earnest workers in this field. Le Filliatre bases his conclusions upon the study of 1,500 cases in which cocaine has been used in the manner suggested, and his series extends over a period of six years. He suggests that from 10 to 30 c.c. of cerebro-spinal fluid should first of all be withdrawn from the theca spinalis. From ½ to 2 c.c. of a 2-per-cent. solution of cocaine should then be injected; the preliminary withdrawal of cerebro-spinal fluid prevents hypertension in the cord and the headache which follows. The author claims for his method freedom from post-anæsthetic vomiting and shock, and from the inevitable risks of general anæsthesia. Other observers, however, whose mode of injecting cocaine differs little in essentials from the above, have recorded unpleasant and even serious after-effects. In straightforward cases the balance of danger to the patient from post-anæsthetic mishaps is, in the opinion of most observers, decidedly in favour of chloroform, ether, and their congeners. The damage caused to the blood elements by the temporary passage through them of these poisons compares favourably with the nervous shock and the paralysis which may follow the most carefully regulated intraspinal injections of cocaine. It is difficult, moreover, to conceive any stage of an operation at which the assistance of its conscious and willing subject could be preferable to that of ordinary helpers; whilst the objections to the probability of the patient seeing and hearing all that takes place around and upon him are obvious. Spinal analgesia is, and will probably remain, infinitely more popular with continental than with British surgeons, if only for the reason that on this side of the Channel the patients' feelings, mental and bodily, are always taken into considerable account. With these observations, Le Filliatre's conscientious advocacy of cocaine as a medium for spinal anæsthesia in cases unsuited to chloroform or ether, is worthy at least of critical study and investigation.

